# Post-injection delirium/sedation syndrome in patients with schizophrenia treated with olanzapine long-acting injection, II: investigations of mechanism

**DOI:** 10.1186/1471-244X-10-45

**Published:** 2010-06-10

**Authors:** David P McDonnell, Holland C Detke, Richard F Bergstrom, Prajakti Kothare, Jason Johnson, Mary Stickelmeyer, Manuel V Sanchez-Felix, Sebastian Sorsaburu, Malcolm I Mitchell

**Affiliations:** 1 Lilly Research Laboratories, Indianapolis, Indiana, USA; 2 F. Bergstrom PK/PD Consulting LLC, Carmel, Indiana, USA

## Abstract

**Background:**

Olanzapine long-acting injection (LAI) is a salt-based depot antipsychotic combining olanzapine and pamoic acid. The slow intramuscular dissolution of this practically insoluble salt produces an extended release of olanzapine lasting up to 4 weeks. However, in a small number of injections (< 0.1%), patients experienced symptoms suggestive of olanzapine overdose, a phenomenon that has been termed "post-injection delirium/sedation syndrome" (PDSS). The authors conducted a series of parallel investigations into the possible reasons PDSS events occur.

**Methods:**

Healthcare providers involved in the PDSS cases were queried for clinical information around the events. Plasma samples from patients experiencing PDSS were collected when possible (12/30 cases) and olanzapine concentrations compared with the known pharmacokinetic profile for olanzapine LAI. Product batches and used vials from the PDSS cases were evaluated for compliance with established manufacturing standards and/or possible user error. Because this depot formulation depends upon slow dissolution at the intramuscular injection site, in-vitro experiments were conducted to assess solubility of olanzapine pamoate in various media.

**Results:**

Injection administrators reported no unusual occurrences during the injection. No anomalies were found with the product batches or the remaining suspension in the used vials. Olanzapine concentrations during PDSS events were higher than the expected 5-73 ng/mL range, with concentrations exceeding 100 ng/mL and in some cases reaching >600 ng/mL during the first hours after injection but then returning to the expected therapeutic range within 24 to 72 hours. Solubility and dissolution rate of olanzapine pamoate were also found to be substantially greater in plasma than in other media such as those approximating the environment in muscle tissue.

**Conclusions:**

Manufacturing irregularities, improper drug reconstitution, and inappropriate dosing were ruled out as possible causes of PDSS. In-vitro solubility and in-vivo pharmacokinetic investigations suggest that PDSS is related to exposure of the injected product to a substantial volume of blood. This exposure is most likely the result of unintended partial intravascular injection or blood vessel injury during the injection (occurring even with proper injection technique) with subsequent seepage of the medication into the vasculature, which would produce higher than intended olanzapine concentrations and symptoms consistent with PDSS.

**Trial Registration:**

ClinicalTrials.gov ID; URL: http://http//www.clinicaltrials.gov/: NCT00094640, NCT00088478, NCT00088491, NCT00088465, and NCT00320489

## Background

Post-injection delirium/sedation syndrome (PDSS), also known as post injection syndrome, is a serious adverse event observed in a small percentage of patients treated with olanzapine long-acting injection (LAI), following approximately 0.07% of injections [1]. Characterized by symptoms related to excessive sedation and/or delirium that occur in temporal proximity to the injection, the syndrome appears consistent with some of the symptoms of oral olanzapine overdose [2]. When the first case was followed by 2 subsequent independent cases close in time, intensive investigations were initiated to understand these occurrences. As additional cases occurred, hypotheses emerged and were evaluated regarding the possible mechanism or mechanisms behind these events.

Because the first such PDSS case revealed unexpectedly high olanzapine concentrations at the time of the PDSS event, it was hypothesized from that first case and those subsequently observed that these events were likely the result of too much olanzapine entering the systemic circulation more rapidly than intended. Olanzapine LAI is composed of an aqueous suspension of a poorly soluble salt (olanzapine pamoate monohydrate). Typical performance is such that almost immediately upon injection of the suspension, a slow dissolution of the olanzapine pamoate monohydrate salt begins at the intramuscular site of injection and systemic olanzapine concentrations are measurable within minutes to hours. Dissolution of the dose then continues slowly over a period of weeks providing sustained, therapeutic systemic concentrations of olanzapine. These concentrations typically reach a peak within the first week after the injection and then gradually decline over the next few weeks, allowing the medication to be injected every 2 to 4 weeks [3]. Similar solubility-limited salt-based depot formulations are known to be advantageous because they provide a slow extended dissolution over a prolonged time while also permitting the dissolution process to begin quickly, allowing for an immediate onset of action from absorption of the disassociated components (in this case, olanzapine and pamoic acid) into the bloodstream [4,5]. For the olanzapine LAI formulation, it is important to note that after every injection except those resulting in a PDSS event, the olanzapine concentration profile does not show any rapid initial release such as a "burst" or a "dose dumping" effect [6] in which a larger amount of the drug is released initially upon injection. Instead, olanzapine concentrations increase slowly after the olanzapine LAI injection, and the slow depot release maintains the olanzapine concentration within a range of approximately 5 to 73 ng/mL (10th percentile for 150 mg/2 weeks to 90th percentile for 300 mg/2 weeks at steady state) [5], which is within the range resulting from within-label oral olanzapine doses [7-9].

Given this pharmacokinetic profile, the occurrence of a single discrete episode of unexpectedly high olanzapine concentrations in some patients soon after intramuscular injection did not appear to have a clear explanation. Hypothesized root causes included product quality issues, errors in reconstitution, inappropriate dosing or administration of the medication, or unanticipated behavior of the formulation under certain physiological conditions, such as accidental intravascular injection. We investigated these potential causes through the following: 1) review of product quality controls, 2) analysis of unused suspension remaining in the product vials of the PDSS cases, 3) review of information from the injection administrators and healthcare personnel involved in the cases for any notable occurrences during the injection process or apparent proximate causes of the event, 4) analysis of plasma samples collected during the PDSS events, and 5) analysis of both the solubility and intrinsic in-vitro dissolution rate of olanzapine pamoate in various media representative of physiological fluids (specifically, blood versus the muscle tissue environment). The key findings from these investigations are described below.

## Method

### Description of PDSS events

A description of the 8 clinical trials and the patient populations on which these analyses are based as well as a description of the first 30 cases of PDSS observed during olanzapine LAI trials are presented by Detke et al. in a companion article [2]. All study protocols were approved by institutional review boards at each site. After receiving a complete description of the study, all patients and/or their authorized legal representatives provided written informed consent before participation.

### Vial and product quality investigation

Manufacturing records including clinical trial lot approval and stability reports were reviewed for those lots of olanzapine LAI involved in PDSS events and for those not involved in PDSS events. The physicochemical properties examined included the crystal form and the particle size (or surface area available for dissolution), which are factors that can affect the rate of olanzapine release if outside the limits specified by the manufacturing process. Manufacturing records were examined to confirm that drug lots met established control standards during manufacturing and to determine whether there were any trends toward smaller particle size, whether particle size changed upon storage, and whether there was homogeneity of particle size distribution from vial to vial.

In addition, when possible, used product vials from the PDSS events were collected for analysis. If an injection resulted in a PDSS event, the healthcare provider was requested to return the leftover vial of reconstituted olanzapine pamoate suspension as well as the leftover diluent vial that had been used to suspend the olanzapine pamoate powder. Vials were analyzed to confirm drug product identity and concentration as well as other product characteristics such as pH, crystal morphology, and particle size.

### Information from clinical sites

Clinical trial investigators provided detailed reports on the PDSS events within 24 hours of the time they occurred. Follow-up was conducted with site personnel either by telephone or during a site visit to gather additional details around the events and to clarify statements in the original event reports. Interactions with site personnel who had been involved in these cases also occurred through various clinical trial meetings and training sessions, allowing for the opportunity to discuss clinical impressions and explore possible proximate causes that may have been apparent in the clinic prior to, during, or after the injection.

### Pharmacokinetic investigations

Plasma samples were collected from blood prospectively in some but not all of the olanzapine LAI clinical trials. After the discovery of the PDSS phenomenon, investigators in all ongoing trials were requested to collect plasma samples within the first 2 hours of onset of the PDSS event and then at approximately 4, 8, 16, 24, and 72 hours after onset or until symptoms resolved. If patients were sent to the hospital for further monitoring, it was requested that such samples be collected by the hospital when possible. Plasma samples were analyzed using validated methodology that included high performance liquid chromatography (HPLC) with electrochemical detection based on Catlow et al. [10] to determine the concentration of olanzapine in each sample. The concentration measurements were conducted by BASi in West Lafayette, IN, USA. Concentrations over time were graphically assessed and compared to the database of olanzapine plasma concentrations from clinical trials that spanned the corresponding range of doses for once daily oral olanzapine and every-2-to-4-week doses of olanzapine LAI [3,8,2].

### Solubility and intrinsic dissolution rate investigations

Two types of investigations were conducted in-vitro to compare the rate of dissolution of olanzapine pamoate monohydrate in various media. The first set of experiments assessed equilibrium solubility by placing an excess of olanzapine pamoate monohydrate in contact with a liquid medium to determine the maximum amount of the salt that could be dissolved per mL of fluid. Materials for the equilibrium solubility experiments included olanzapine pamoate monohydrate; human plasma (pH = 7.67) from Biological Specialty Corp, Colmar, PA; USP pH 7.68 phosphate buffer; USP pH 6.80 phosphate buffer; and plasma ultrafiltrate (plasma passed through a 10,000 molecular weight filter in order to remove proteins and lipids). Human plasma was the primary medium of interest and was used to understand whether the solubility of olanzapine pamoate changes if it comes into contact with substantial quantities of blood. The pH 7.68 buffer was selected because it had the same pH as the plasma lots used, and the pH 6.80 buffer was used as a reference medium because this buffer had been used in prior in-vitro dissolution studies done as part of the preclinical development for olanzapine pamoate [unpublished data]. These aqueous buffers were used as a proxy for the extracellular fluid of muscle tissue. The purpose of including the plasma ultrafiltrate was to assess the importance of proteins and lipids present in the plasma upon the solubility and dissolution of olanzapine pamoate and to be a further surrogate or proxy for the extracellular fluid in muscle tissue.

For the plasma and plasma ultrafiltrate equilibrium solubility in-vitro experiments, 8 mg olanzapine pamoate monohydrate was placed in a vial and then the appropriate medium was added; for the phosphate buffer analyses, 2 mg of olanzapine pamoate monohydrate was used. The samples were placed into a heated 37°C precision water bath and then shaken continuously. Liquid sample fractions were obtained after approximately 24 and 48 hours. The samples were centrifuged at 5000 rpm for 10 minutes prior to their preparation for analysis. Analysis of samples was conducted in triplicate for each medium. Olanzapine concentrations were determined by liquid chromatography-mass spectrometry.

The second type of experiment assessed intrinsic dissolution rate (IDR), which measures the rate at which olanzapine pamoate dissolves by exposing a constant surface area of a compressed pellet of the salt to sink condition for the various media. Materials for the IDR experiment were olanzapine pamoate monohydrate, human plasma (pH = 7.46) from Biological Specialty Corp, Colmar, PA, and USP pH 7.46 phosphate buffer. Air was removed from the phosphate buffer by purging with helium for 5 minutes and from the plasma by sonification for 5 minutes. For each assessment, 100 mg of olanzapine pamoate monohydrate was compressed using a Carver Press at a pressure of 3000 pounds for 1 minute to produce a pellet of the salt with a surface area of 0.5 cm^2^. The pellet was placed into 500 mL of the media in a dissolution bath using a Wood's apparatus set to a rotation of 100 rpm at a temperature of 37°C. Samplings of 1 mL per time point were collected at 1, 5, 10, 15, 20, 30, 45, 60, 120, and 180 minutes, and the olanzapine pamoate concentration in each sample was analyzed using liquid chromatography-mass spectrometry. The IDR experiments for each of the media (plasma and aqueous buffer) were performed in triplicate. The integrity of the pellet was checked visually to establish whether a constant surface area was maintained during the experiment.

## Results

### Vial and product quality analyses

All drug lots met established standards during their manufacturing. Approval and stability data for the drug lots involved in PDSS cases were comparable to data from other clinical trial lots in which PDSS was not observed. Clinical trial lot data used to approve the lots for clinical use indicated that there have been no lots with significant amounts of small particles. The particle size distributions for the lots involved in the PDSS cases were consistent with the particle size distributions tested in a clinical pharmacology study (Study F1D-EW-LOBS, NCT00094640) and within the limits specified by the manufacturing process, within which there is no impact on the pharmacokinetic profile of olanzapine LAI. Furthermore, the particle size distribution of these drug lots did not change upon storage, and homogeneity of the drug product particle size distribution from vial to vial was demonstrated.

Eleven used olanzapine pamoate vials and 10 used diluent vials were collected from the PDSS cases. The residual suspension in all 11 olanzapine pamoate vials exhibited the expected physicochemical properties (i.e., potency, related substances, pH, particle size, and crystal morphology). The 10 returned diluent vials were all confirmed to be the appropriate diluent. These analyses indicated no evident errors in drug reconstitution by the site and no product quality issues.

### Follow-up information from clinical investigators

Healthcare personnel involved in the PDSS events did not report any difficulties or peculiarity with the administration of the injection itself. They did not observe significant blood return at the site of the injection either upon aspiration of the syringe prior to injection or following the injection, other than what would be considered typical, nor did they note any hematoma or other anomalies at the site of injection either before or after the injection. Questioning of the site personnel revealed that the injection administrators were typically nurses with significant clinical experience in performing intramuscular gluteal injections, although there was a range of experience noted and also in some cases the physician performed the injection. Investigators did not report variation from the usage of the 1.5-inch (or 35 mm) 19-gauge needle supplied with the medication. Although a 2-inch (or 50 mm) needle was also available for use with obese patients, needle length did not appear to be a factor in the events. Analysis of the anecdotal reports from the sites regarding specific cases did not identify any common factor among the cases that could be viewed as a potentially proximate cause of the event.

### Pharmacokinetic analyses

**Figure 1:**
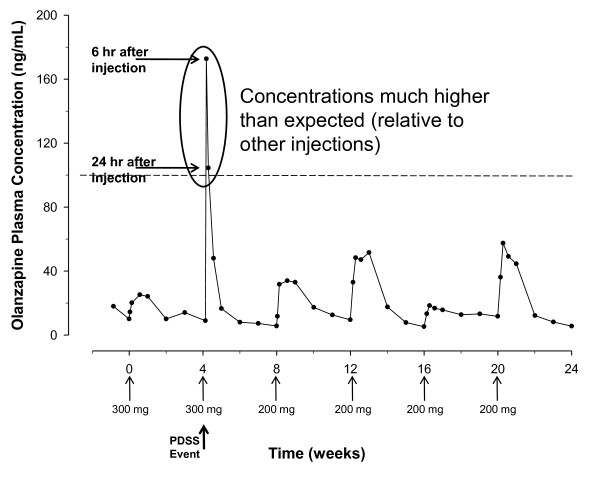
**Olanzapine plasma concentrations across multiple injections in a patient with a PDSS event**. The figure illustrates the olanzapine plasma concentration profiles after 6 different olanzapine LAI injections in one patient who experienced a PDSS event at the second injection. Arrows below the x-axis indicate injections. Higher than expected olanzapine plasma concentrations were measured at 6 and 24 hours after the second injection, with concentrations returning to the expected therapeutic range after 48 hours. Olanzapine concentrations at subsequent injections remained in the expected therapeutic range. The dashed line indicates 100 ng/mL; all of the assessed PDSS cases had maximum olanzapine concentrations higher than this value.

Figure 1 presents olanzapine plasma concentrations for the first case of PDSS [2], which occurred during the conduct of a pharmacokinetic study [11], allowing for analysis of olanzapine concentrations over the course of 6 monthly injections. The patient (a male smoker) received his first injection of olanzapine LAI at a dose of 300 mg/4 weeks. The pharmacokinetic profile for this first injection was generally consistent with the typical profile at this dose, with the patient's olanzapine concentrations over the dosing interval averaging approximately 17 ng/mL. Forty-five minutes after the second injection, the patient experienced severe sedation as well as other symptoms of PDSS, including disorientation, dizziness, weakness, and tension in the legs. The patient slept and then had a blood sample drawn at approximately 6 hours after injection that revealed an olanzapine concentration of 172.75 ng/mL, which was above the expected range for this patient or this dose. At that 6-hour time point, the patient felt better but remained sleepy. Olanzapine concentrations returned to a normal range over the next 24 to 48 hours, and all symptoms of PDSS had resolved by 24 hours after injection. The patient continued in the trial, although at a lower dose (200 mg/4 weeks), receiving 4 more injections as per protocol, with no further PDSS events and with olanzapine concentrations that were generally consistent with other patients at this dose level.

**Figure 2:**
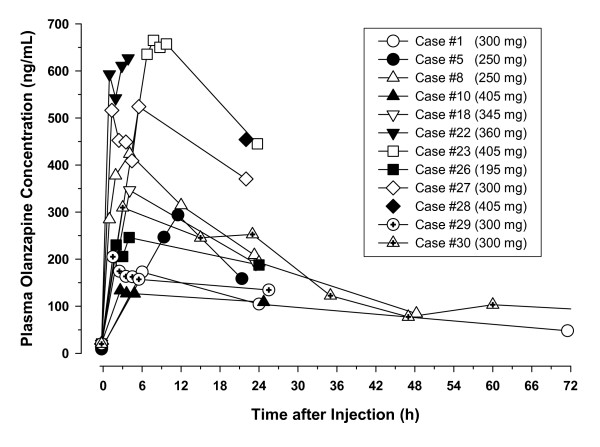
**Olanzapine plasma concentrations observed over time in PDSS events**. The figure shows olanzapine plasma concentrations from the time of the injection associated with the PDSS event up to 72 hours after that injection. Olanzapine plasma concentration values plotted at time 0 hour (pre-injection) that anchor the concentration curves are either based on the patient's data for measurements made before other injections or are presumed to be approximately 20 ng/mL based on the general population's typical pre-injection concentration. Only the data after injection (samples collected beyond 0 hour) are actual measurements for samples collected for these events. Case numbers correspond to the cases presented in Detke et al [2].

**Figure 3:**
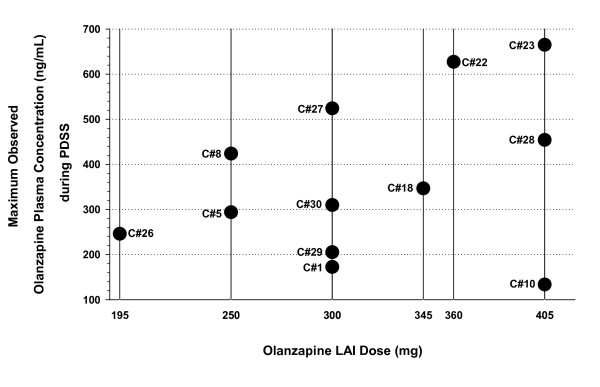
**Maximum observed olanzapine plasma concentration by dose during the PDSS events**. The figure illustrates the maximum observed olanzapine plasma concentration measured during the PDSS events by dose. C# = case number. Case numbers correspond to the cases presented in Detke et al [2].

Plasma samples were collected for a total of 12 of the 30 PDSS injection events. Figure 2 illustrates the olanzapine plasma concentrations measured over time during these events. In all 12 cases, observed olanzapine concentrations exceeded the expected range of concentrations for these doses. Because there were only a limited number of samples obtained during and after an event, it is not known that these were the highest or peak olanzapine concentrations during the event. However, the concentration pattern from these data demonstrated a substantial increase in olanzapine concentrations to supratherapeutic levels in the hours after the injection, followed by a gradual return to typical levels over the next 24 to 72 hours, concordant with the resolution of the event's clinical symptoms. Figure 3 presents the maximum observed olanzapine plasma concentrations during the events by dose. There was not a consistent relationship between maximum olanzapine concentration measured during the event and dose injected, suggesting that PDSS events can occur after giving any olanzapine LAI dose and that dose size is not a principal factor.

Finally, while the olanzapine plasma concentrations in the hours immediately following the onset of a PDSS event were substantially elevated, appropriate therapeutic concentrations were maintained for the remainder of that injection interval. Therefore, despite the apparently early and excessively fast release of a portion of the olanzapine LAI dose, some portion of the dose appeared to continue to provide a slow and sustained release of olanzapine over a period of weeks, consistent with the expected performance characteristics of a depot.

### Solubility and intrinsic dissolution rate investigations

Table 1 reports the equilibrium solubility results for olanzapine pamoate in various media. Results from the 24- and 48-hour tests were similar. However, there was somewhat less variability between replications at the 48-hour time period suggesting that the 48-hour results approach equilibrium. At the 48-hour sampling time, mean solubility of olanzapine pamoate in plasma (0.986 mg/mL) was established to be substantially higher than in other media studied, including plasma ultrafiltrate (0.176 mg/mL); phosphate buffer pH 7.68 (0.060 mg/mL); and phosphate buffer pH 6.80 (0.016 mg/mL). The pH 7.68 buffer results were in excellent agreement with and replicated historical solubility results [unpublished data], thus suggesting that any differences in procedures used in the current solubility studies did not introduce a bias.

**Table 1:** Solubility of olanzapine pamoate monohydrate in various media determined at 24 and 48 hours (37°C)

Medium (time in hours)	Solubility (mg/mL)	Mean Solubility (mg/mL)
Plasma (24 hr)	0.986	0.918
Repeat #1	0.935	
Repeat #2	0.832	

Plasma (48 hr)	1.090	0.986
Repeat #1	0.890	
Repeat #2	0.978	

Plasma ultrafiltrate^a ^(24 hr)	0.150	0.187
Repeat #1	0.236	
Repeat #2	0.176	

Plasma ultrafiltrate (48 hr)	0.160	0.176
Repeat #1	0.161	
Repeat #2	0.207	

pH 7.68 buffer (24 hr)	0.058	0.052
Repeat #1	0.053	
Repeat #2	0.044	

pH 7.68 buffer (48 hr)	0.072	0.060
Repeat #1	0.063	
Repeat #2	0.046	

pH 6.80 buffer (24 hr)	0.024	0.013
Repeat #1	0.007	
Repeat #2	0.008	

pH 6.80 buffer (48 hr)	0.016	0.016
Repeat #1	0.012	
Repeat #2	0.021	

^a^Plasma ultrafiltrate (i.e., plasma filtered for proteins and lipid particles) was used to assess the importance of the presence of proteins and lipids upon solubility and dissolution of olanzapine pamoate monohydrate

The intrinsic dissolution rate experiments indicated that the rate of dissolution of olanzapine pamoate in plasma (0.73 mg/hr·cm^2^) was approximately 6 times higher than in the phosphate buffer (pH 7.46 = 0.12 mg/hr·cm^2^). The faster rate of in-vitro dissolution in plasma is consistent with the finding of higher solubility in plasma and consistent with the established Noyes-Whitney theory predicting that for a constant surface area, the intrinsic rate of dissolution increases with an increase in the equilibrium solubility of the solute in the medium [12,13].

## Discussion

Olanzapine pamoate is an insoluble salt-based depot formulation that is designed to release olanzapine slowly at the site of the intramuscular gluteal injection over the course of several weeks. The occurrence of a small number of events marked by excessive sedation and/or delirium occurring within 1 to 3 hours after the injection, typically within the first hour [2], resulted in our investigating the possible reason(s) for these occurrences. Those investigations did not uncover any evidence suggesting manufacturing irregularities or human error as possible causes of these events. Instead, the converging evidence indicates that this post-injection syndrome, with its clinical presentation consistent with olanzapine overdose, is related to a more rapid than intended dissolution of a portion of the olanzapine LAI dose in the hours soon after the injection, resulting in higher than intended olanzapine plasma concentrations during the post-injection period. This inadvertent early release of olanzapine can occur if olanzapine LAI comes into contact with a substantial volume of blood. Intended for intramuscular injection only, olanzapine LAI could potentially come into contact with blood through various mechanisms but most likely as a result of accidental intravascular injection or blood vessel injury during the intramuscular injection process.

### Pharmacokinetic analyses

#### Excessive olanzapine concentrations

Although the olanzapine plasma concentration data obtained during PDSS injection events were sparse, the pattern emerging from these data indicated the presence of excessive concentrations of olanzapine in the hours immediately after the injection. These findings parallel the clinical findings [2], which indicated symptoms consistent with olanzapine overdose. Moreover, the timing of the symptoms and their resolution appeared to correspond to the concentration-time profile, with symptoms resolving and olanzapine concentrations returning to the expected range within 24 to 72 hours.

#### No clear correlation with dose

Maximum olanzapine concentrations during the event did not appear to correlate with dose. Although olanzapine pharmacokinetic characteristics are associated with wide interpatient variability in olanzapine plasma concentrations, an evaluation of the available pharmacokinetic databases for various formulations of olanzapine indicate that plasma concentrations for olanzapine generally demonstrate dose-proportional increases [14]. However, results for the PDSS cases found no consistent pattern across cases. For example, the lowest observed PDSS peak olanzapine concentration occurred after administration of the largest olanzapine LAI dose (405 mg), suggesting that only a portion of the dose was released into systemic circulation prematurely. Also, PDSS events have occurred after doses of 195 to 405 mg olanzapine LAI doses, representing nearly the full spectrum of therapeutic dose strengths (150 to 405 mg). Thus, the variability in observed olanzapine concentrations for the PDSS cases most likely relates more strongly to the portion of the dose that prematurely enters the systemic circulation rather than to the total dose injected.

### Solubility

**Figure 4:**
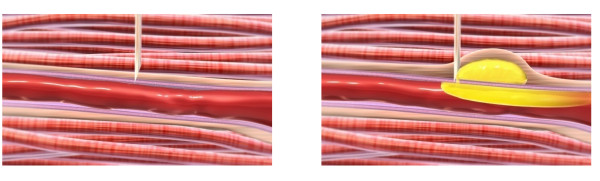
**Illustration of proposed mechanism for olanzapine LAI distribution (in yellow) after vessel damage by nicking**. The figure illustrates the proposed mechanism for distribution of the olanzapine LAI suspension during a PDSS event. The first panel depicts the tip of the syringe needle piercing the wall of the blood vessel situated within the muscle bed. In the second panel, the medication (in yellow) has been injected into the muscle tissue and is leaking into the blood vessel through the punctured vessel wall.

The solubility of olanzapine pamoate monohydrate in plasma was shown to be substantially higher than in aqueous media approximating the extracellular fluid in muscle tissue. Greater solubility of olanzapine pamoate in blood than in the fluid bathing the muscle tissue affords the basis for a theory as to the mechanism leading to the observed excessive systemic olanzapine concentrations associated with PDSS events. Because of this greater solubility, a faster rate of dissolution of the pamoate salt would occur as a result of contact between the injectabl esuspension of drug product, olanzapine LAI, and a substantial quantity of blood. That is, if olanzapine pamoate monohydrate were exposed to a sufficiently large volume of blood, an amount of olanzapine that approaches the equilibrium solubility of the salt could dissolve. Under static conditions, a hematoma consisting of 20 mL blood would appear to be required for the dissolution of approximately 20 mg olanzapine (based upon the 0.986 mg/mL estimate for equilibrium solubility of olanzapine pamoate in plasma). This amount of olanzapine is a widely used oral dose, and would not be expected to lead to profound sedation, even if that amount were to gain rapid access into the bloodstream. Moreover, a hematoma much larger than this would likely be detectable by the patient or clinician. Therefore, a more plausible theory is that in order to dissolve the amount of olanzapine needed to achieve the observed concentrations and to produce the observed clinical effects, the olanzapine LAI would need to be exposed to a substantial amount of blood, the volume of which would likely require a continuous flow. Blood flow would also aid dissolution of the olanzapine pamoate through the motion or agitation in the bloodstream. Thus, a substantial amount of olanzapine could be rapidly dissolved even if only a portion of the olanzapine LAI dose were accidentally injected into a blood vessel or if the needle accidentally nicked or pierced a proximal vessel during the injection process, providing a track to access the bloodstream (Figure 4).

### Probable mechanism

Accidental intravascular injection of drugs intended for intramuscular injection is a recognized risk of this route of administration [15-20], and there is evidence that intramuscularly injected medications can enter the vasculature [21]. Although not all accidental intravascular injections would necessarily produce noticeable symptoms, one intramuscularly injected medication with a clinically distinguishable and well characterized post injection syndrome is penicillin procaine G [16,22]. This procaine-penicillin salt can produce a reaction known as Hoigne's syndrome following accidental intravascular injection [16] as the salt rapidly dissolves in blood, allowing free base procaine to penetrate the brain in excessive amounts and causing mental confusion. As occurs with the penicillin-procaine salt, direct entry of the olanzapine pamoate salt into the bloodstream would substantially enhance its rate of dissolution, especially under the physiological conditions in which a continuous flow of blood occurs. Continuous blood flow could promote the release of a substantial amount of olanzapine over a period of minutes to hours.

It should be noted that a direct intravascular injection of the full dose of olanzapine LAI would be difficult to achieve. The features of the delivery device (i.e., a 19-gauge needle) and the properties of the olanzapine LAI injection (i.e., a viscous suspension of solid material having a total injection volume between 1.5 to 3.0 mL), impose physical limitations that make accidental intravascular injection of the full dose highly improbable. The needle diameter and long bevel make accidental cannulation of a blood vessel highly unlikely, and such placement of the needle would results in blood aspiration before giving the injection. Nonetheless, even if the needle were accidentally positioned completely inside a blood vessel, the injection of up to 3.0 mL of the olanzapine LAI suspension would likely obstruct blood flow through that vessel and only result in a portion of the dose going into the circulation. Considering the needle size, the injection volume, the physical properties of the suspension of olanzapine pamoate, and prescribed intramuscular injection technique, it is likely that only a portion of the olanzapine LAI dose can accidentally be injected into the bloodstream. These physical limitations to a full intravascular injection are consistent with the pharmacokinetic findings, which suggest that only a portion of the dose enters the vasculature prematurely.

Ultimately, the potential mechanisms by which the suspension comes into contact with blood could be various. For instance, direct injection into a blood vessel may be one method, but nicking a blood vessel during the injection process (Figure 4), pooling of a large quantity of blood at the site of the drug deposit in the muscle, or injection into a rich capillary bed could also be possibilities. Although proper intramuscular injection technique is important to minimize the risk of a PDSS injection event, the PDSS events still occurred even when proper technique was being used. Proper injection technique would only avoid placement of the tip of the needle directly inside a vessel, but other forms of blood vessel involvement could still occur. For example, if the needle, when inserted, passed completely through a blood vessel, aspiration of the syringe would not necessarily yield blood because the tip of the needle would no longer be located in the blood vessel. The injection administrator would then inject the bolus of medication into the muscle beyond or in near proximity to the punctured blood vessel. However, when the needle was withdrawn, the medication could then track back and enter the blood vessel at the site of the puncture. Therefore, a negative finding upon aspiration is not necessarily indicative that the medication has not or will not come into contact with blood. This explanation would be consistent with the reports by the injection administrators that they had used correct injection technique at the time of the events. It would also be consistent with the finding that the PDSS rate of occurrence remained consistent over the course of the clinical trials despite additional injection technique training for the clinical sites. This consistency of PDSS rate over time, despite further training and increased vigilance with regard to injection technique, suggests that this rate (0.07% of injections) may reflect the naturally occurring background rate of accidental blood vessel contact (puncture or nicking) during intramuscular injections. This conjecture appears to be further supported by the finding of a very similar rate (0.08% of injections) for the penicillin procaine G post-injection reaction known as Hoigne's syndrome [16], which is also presumed to occur through the mechanism of inadvertent intravascular injection, irrespective of good injection technique.

### Proposed mechanism and timing of onset of PDSS

One seemingly contradictory finding would appear to be the timing of onset of the clinical symptoms of PDSS. Symptoms of accidental intravascular injection of medications are often assumed to be nearly instantaneous following injection (as is the case with dental anesthetics). However, for olanzapine LAI, data instead suggest that the increase in olanzapine systemic exposure during a PDSS injection event progressed over a period of hours. This course also corresponds to the observed clinical course [2], in that symptoms in some cases took an hour or more to appear, and in almost all cases, symptoms of the syndrome gradually evolved over the course of minutes to hours rather than seconds. This progression is likely attributable to the fact that the olanzapine pamoate monohydrate salt must first dissolve and then dissociate into its components, olanzapine and pamoic acid. Thus, even when there is substantial contact between the suspension and blood, complete dissolution of olanzapine LAI will not occur immediately. Consequently, the rate of change in systemic olanzapine concentration is much less rapid, changing over a period of hours during a PDSS event rather than the almost instantaneous increase that would occur if a solution of olanzapine base (as opposed to olanzapine pamoate salt) were to be injected intravenously or even intramuscularly. Although the increase in olanzapine concentrations during a PDSS event is still much faster than intended for this slow-release depot formulation, the fact that this premature release occurs more gradually than a direct injection of an olanzapine solution may also account for the lack of cardiorespiratory depression seen in any of the PDSS cases to date [2].

There has also been a wide variability in the timing of onset of PDSS symptoms, with onset times ranging from immediately after the injection to as late as 3 or more hours after injection. The length of the delay between time of injection and time of first PDSS symptoms is likely dependent on a number of factors, including the size of the affected blood vessel, the degree of vascular injury, the volume and rate of blood flow at the site of injury, the amount of olanzapine pamoate coming into direct contact with a substantial quantity of blood, and perhaps patient-specific factors such as clotting speed. It is therefore the variable rate of dissolution of olanzapine LAI under the variable physical conditions or situations leading to a PDSS injection event that likely influences not only the time of onset but also the intensity of the adverse events associated with a PDSS injection event. Given the potential for variability in the confluence of the many factors impacting the time course of a PDSS injection event, it is possible that an event could begin soon after injection (e.g., if a major blood vessel received a larger injury) or could be delayed for hours (e.g., if a smaller blood vessel received a smaller injury, if a small injury during the injection process were later exacerbated by additional physical factors, or if the distance along the injection track between the injury and the deposited medication were longer).

As for whether other mechanisms could explain PDSS events, any explanation would need to account for the elevated systemic olanzapine concentrations during the event. Therefore, the mechanism must entail the dissolution and absorption of a greater portion of the dose initially than is expected for this depot formulation. It is possible that this early and time-limited increase in solubility could be accounted for by other means or that excessive contact between olanzapine pamoate and blood could occur through means other than accidental blood vessel injury during the injection process. Although there was no indication that massage of the injection site, muscle injury, or high-pressure injection was involved with any of the events, we cannot definitively rule these out as contributors to the events. With regard to massage at the injection site, current administration instructions state that massage of the injection site should not be performed. Also, typical injection forces for olanzapine LAI are low. In an in-vitro compression test, typical injection forces were measured to be approximately 2 pounds of force, which in our testing was less than that required to inject other depot medications. In the event of a needle clog, it is possible that the injection force could be higher; however, there has been no evidence to suggest that high injection pressure was a contributing factor in any of the events seen to date. Further research would be needed to develop and/or confirm other possible explanations.

## Conclusions

All existing data point to accidental contact between olanzapine LAI and blood as the proximate cause of PDSS. Patients experiencing PDSS events had demonstrably higher than expected systemic concentrations of olanzapine only at the time of the PDSS event. The pharmacokinetic profile during these events indicated a more rapid release of olanzapine than normally intended during the hours immediately after the injection but with concentrations returning to expected levels for the remainder of the injection interval. Product quality issues and administrator error were ruled out as possible causes. Because olanzapine pamoate is substantially more soluble in blood than in the fluid bathing the muscle tissue and consequently dissolves more rapidly in blood, the most likely explanation for PDSS is that a portion of the intramuscularly injected olanzapine pamoate dose accidentally enters the bloodstream as a result of injury to a blood vessel during the injection process, effectively resulting in intravascular injection of a limited portion of the olanzapine LAI dose.

## Competing interests

All co-authors except RFB are employees and/or shareholders of Eli Lilly and Company. RFB was an employee of Eli Lilly and Company at the time of these investigations.

## Authors' contributions

DPM provided medical leadership for the investigations of the PDSS events and was responsible for study design and data collection. HCD was responsible for study design and data collection and was responsible for drafting of the manuscript. RFB led the pharmacokinetic investigations. PK was responsible for the pharmacokinetic analyses. JJ was responsible for the solubility analyses. MS was responsible for the product quality analyses. MSF was responsible for solubility and IDR design. SS was responsible for data collection. MIM was responsible for study design and data collection for the clinical pharmacology and biopharmaceutical studies. All authors contributed to the analysis and interpretation of the data and reviewed and approved the final version of the manuscript.

## Pre-publication history

The pre-publication history for this paper can be accessed here:

http://www.biomedcentral.com/1471-244X/10/45/prepub
